# Promoting Barrier Performance and Cathodic Protection of Zinc-Rich Epoxy Primer via Single-Layer Graphene

**DOI:** 10.3390/polym10060591

**Published:** 2018-05-28

**Authors:** Jingrong Liu, Tao Liu, Zhangwei Guo, Na Guo, Yanhua Lei, Xueting Chang, Yansheng Yin

**Affiliations:** College of Ocean Science and Engineering, Shanghai Maritime University, Shanghai 201306, China; liujingrong@stu.shmtu.edu.cn (J.L.); guozhangwei891212@163.com (Z.G.); naguo@shmtu.edu.cn (N.G.); xtchang@shmtu.edu.cn (X.C.); ysyin@shmtu.edu.cn (Y.Y.)

**Keywords:** graphene, zinc-rich epoxy primer, cathodic protection, localized electrochemical impedance spectroscopy

## Abstract

The effect of single-layer graphene sheets (Gr) on the corrosion protection of zinc-rich epoxy primers (ZRPs) was investigated. Scanning electron microscopy (SEM) with an energy dispersive spectrometer (EDS) were used to characterize morphology and composition of the coatings after immersion for 25 days. The cross-sectional SEM images and X-ray photoelectron spectroscopy (XPS) confirmed that the addition of single-layer graphene facilitated assembling of zinc oxides on the interface between the coating and the steel. The open circuit potential (OCP), electrochemical impedance spectroscopy (EIS) measurements revealed that both the cathodic protection and barrier performance of the ZRP were enhanced after addition of 0.6 wt. % Gr (Gr0.6-ZRP). In addition, the cathodic protection property of the Gr0.6-ZRP was characterized quantitatively by localized electrochemical impedance spectroscopy (LEIS) in the presence of an artificial scratch on the coating. The results demonstrate that moderate amounts of single-layer graphene can significantly improve corrosion resistance of ZRP, due to the barrier protection and cathodic protection effects.

## 1. Introduction

Zinc-rich primers (ZRPs) are some of the most widely used and effective materials for protecting steels against corrosion [1,2,3]. Their anticorrosion properties mainly depend on two protective mechanisms when the electrolyte penetrates into the ZRPs [4,5,6,7]. First, ZRPs provide cathodic protection by sacrificial anodic dissolution, while in electrical contact with the steel substrate. Second, ZRPs also exhibit barrier protection by forming a stable layer of zinc oxidation products. These products fill the pores of the coating and act as an insulating barrier that stops the electrolyte from reaching the steel surface [8]. It has been reported that cathodic protection is only active for a short period of time due to the loss of the electrical contact between the spherical zinc particles and steel substrates [9,10]. Therefore, high zinc content (normally higher than 65 wt. %) is necessary to ensure electrical conductivity [11]. Nevertheless, the utilization ratio of the zinc particles is very low, due to the isolated effect of the non-conductive binders [12]. In addition, the low utilization of zinc powder results in environmental pollution and is a waste of resources.

Efforts have been made to improve the protective efficiency of ZRPs by utilizing different types of electrical conducting additives, e.g., polypyrrole, polyaniline, Al pigments, nano clay, carbon black, and carbon nanotubes [5,6,7,9,13]. However, these additives usually offer only a single protective effect: they provide either cathodic protection or barrier protection. Recently, an attractive option for improving the performance of ZRPs by utilizing graphene (Gr) was discussed, which has unique properties that are principally attributed to its 2D hexagonal lattice structure [14]. Gr has excellent electrical and mechanical properties, a high surface area, high-energy barrier, chemical inertness, and small geometric pores [15,16]. Gr can act as a “bridge” between the zinc particles and steel substrate due to its high electrical conductivity (2.5 × 10^5^ cm^2^ V^−1^ s^−1^) [17]. Additionally, Gr offers exceptional barrier protection for steels, even after electrolyte penetration [18].

To date, the majority of work investigating the cathodic protection of ZRPs relied on the salt spray tests. Actually, the methods can only show the apparent changes for the coating. The OCP is also a good test method for evaluation of the cathodic protection degree of the ZRPs. However, potential variation is not able to reflect the local impedance changes at the area where zinc particles provide cathodic protection. The application of suitable evaluation techniques is important for optimizing the protection performance of a coating. In this regard, localized electrochemical impedance spectroscopy (LEIS) can be used as a powerful tool to provide enough insight on the protection mechanisms of these coatings, especially in the presence of artificial damage scratch.

## 2. Materials and Methods

### 2.1. Modified Graphene Preparations

Covalent [19] and noncovalent [20] methods have been extensively researched to improve the solubility of Gr. Because covalent functionalization may damage the structure of Gr, a noncovalent functionalization by sodium dodecyl benzene sulfonate (SDBS) was employed to improve the solubility of Gr in this work [21]. Gr was provided by Angstron Materials Co. (Dayton, OH, USA). SDBS (0.2 mg mL^−1^, Aladdin Co. Shanghai, China) was added into 0.1 mg mL^−1^ Gr with 200 mL deionized water and dispersed for 5 h using an ultrasonic method. The black precipitate was filtered via vacuum filtration and washed with deionized water. Then, the modified Gr (SDBS-Gr) was dried at 100 °C for 24 h and stored for further use (Figure S1).

### 2.2. Coating Sample Preparation

Epoxy resin (E44) and polyamine hardener (T31) were purchased from Sanmu Group Co. (Guangzhou, China). The zinc particles, with average diameters of 3–5 μm, were provided by Sinopharm Chemical Reagent Co. (Shanghai, China). Coatings A (ZRPs) was prepared containing 80 wt. % of the zinc particles. Coatings B (Gr0.6-ZRPs) had modified Gr contents of 0.6 wt. %. The specifications of the different coatings are listed in Table 1.

To prepare the coatings, the epoxy resin was mixed with modified Gr while being stirred at 2000 rpm for 5 h at room temperature. The mixtures were kept in a vacuum chamber for 60 min to remove bubbles and then sonicated for 60 min. The zinc powders were added to the mixtures and stirred for 60 min. Finally, a stoichiometric amount of hardener with a weight ratio of 4:1 (epoxy resin: hardener) was added, and the mixture was stirred.

Prepared coatings were air sprayed on DH32 steel (supplied by Baosteel Inc., Shanghai, China) with the following chemical composition (wt. %): 1.52% Mn, 0.7% Ni, 0.25% Cu, 0.04% Al, 0.15 Cr, 0.055% C, and 97.2% Fe.

The dimensions of each specimen were 2 cm × 2 cm × 0.3 cm. Prior to coating, the specimens were ground sequentially with silicon carbide papers ranging from 200 to 800 grit, degreased with acetone, washed with distilled water, and air dried. The coated samples were kept at 25 °C for seven days. The dry film thickness was controlled at around 100 μm.

### 2.3. Characterization of the Sample Morphology and Composition

After 25 days of immersion in a 3 wt. % NaCl solution, the samples were removed from the test baths, washed with deionized water three times, and fully dried in high-purity (99.999%) nitrogen. The surface morphology and cross-sectional views of the coated samples were observed via SEM (JEOL JSM-7500F, Tokyo, Japan). The elemental composition was obtained through coupled EDS. After immersion for 25 days, the cross-sectional compositions of the samples were analyzed using XPS (AXIS UltraDLD, Tokyo, Japan). The wide-range XPS results (0–1200 eV at an emission angle of 45°) of the binding energy spectra and XPS high-resolution spectra of C 1s scans were recorded.

### 2.4. Electrochemical Measurements

Electrochemical measurements were conducted on the coated samples using a standard three-electrode cell, where a platinum (Pt) plate and a saturated calomel electrode (SCE) were used as the counter electrode and reference electrode, respectively. Prior to obtaining the EIS measurements, the electrode was immersed in the test solutions for 2 h, allowing a steady-state open-circuit potential (OCP) to be achieved. The EIS was measured at OCP using an AutoLab electrochemical workstation (Metrohm Autolab, Zurich, Switzerland). The measuring frequency was between 10^−2^ and 10^5^ Hz, with a sine wave disturbance amplitude of ±10 mV. The impedance data were fitted using the software program Zsimpwin (E Chem Software, Ann Arbor, MI, USA).

The LEIS measurements were used to study the cathodic protection properties of the Gr-ZRPs in the presence of an artificial defect (0.2 mm × 1 mm) using an M470 scanning electrochemical workstation (Bio-logic, Seyssinet-Pariset, France). A three-electrode cell was used with a saturated calomel electrode as the reference electrode, a carbon rod as the counter electrode, and the coated sample as the working electrode. A Pt microprobe with a 5-μm tip was utilized during the LEIS measurements. The distance between the electrode surface and the probe tip was adjusted to 100 μm, and the scanning area was 2 mm × 1 mm. The LEIS measurements were carried out with a current amplitude of 10 μA and at a frequency of 10 Hz. The testing electrolyte was a 5 mM NaCl solution with a low electrical conductivity to ensure accuracy of the results [6].

The open circuit potential (OCP) measurements were also performed to evaluate the cathodic protection behavior of zinc rich coatings for 600 h of immersion. After 24 h of immersion, the coatings were artificially scratched to accelerate testing. 

## 3. Results and Discussion

**Figure 1:**
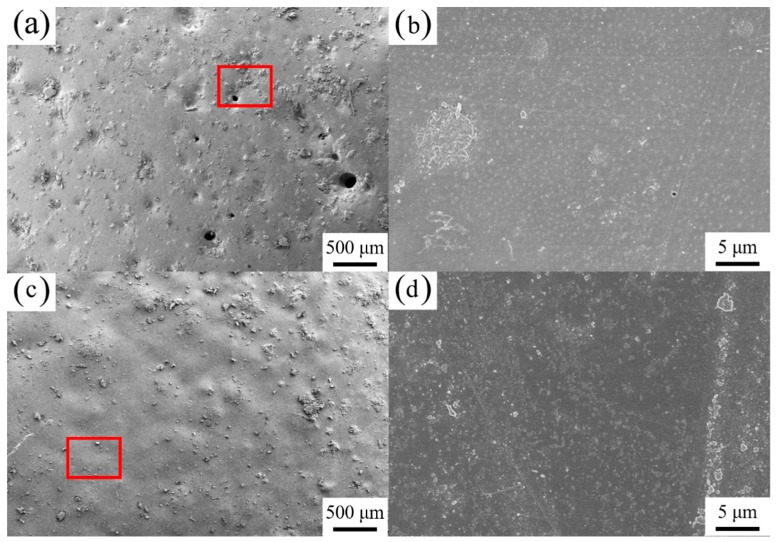
The SEM images for the coated samples (**a**,**b**) ZRPs and (**c**,**d**) Gr0.6-ZRPs after immersion in a 3 wt. % NaCl solution for 25 days, where photos with two magnifications are provided (the areas marked by the red box are measured by EDS mapping in Figure 2).

**Figure 2:**
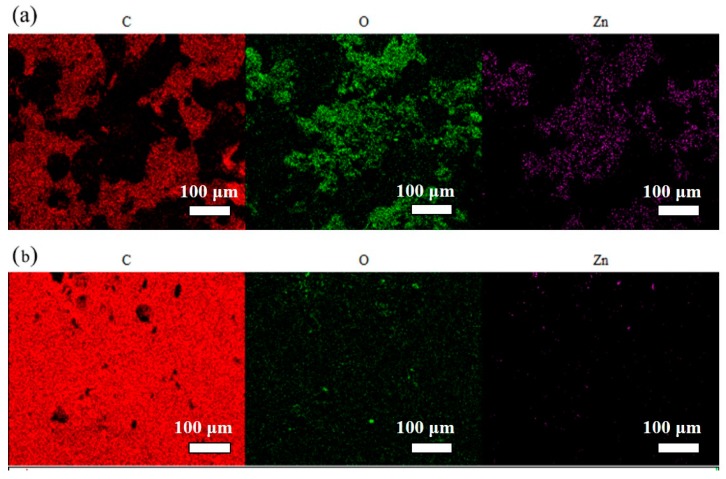
The EDS mapping and spectra for the coated samples (**a**) ZRPs and (**b**) Gr0.6-ZRPs after immersion in a 3 wt. % NaCl solution for 25 days (carbon with red, oxygen with green, and zinc with purple).

Figure 1 shows surface-view SEM images for the coatings A and B after exposure to the NaCl solution for 25 days. Some pores and defects can be observed on the surface of the coating A, which indicates the coating A degraded after immersion of 25 days. However, the surface B is smooth and uniform with addition of 0.6 wt. % of single-layer Gr. In addition, numerous zinc oxygen products were presented on the coating A, which was characterized by EDS spectra in Figure 2a. In the presence of 0.6 wt. % of single-layer Gr, few zinc elements can be observed in the spectra, as shown in Figure 2b. Figure S2 provides a TEM morphology of single-layer Gr used in the current study. 

**Figure 3:**
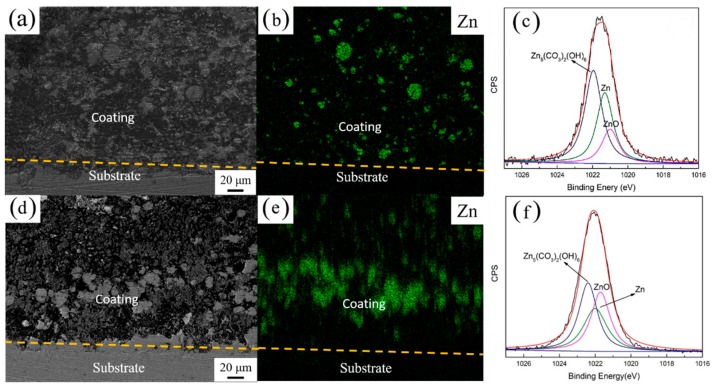
The cross sectional SEM images and Zn 2p3/2 high-resolution XPS spectrum for the coated samples (**a**–**c**) ZRPs and (**d**–**f**) Gr0.6-ZRPs after immersion in a 3 wt. % NaCl solution for 25 days.

Figure 3 shows the SEM cross-sectional views with the corresponding EDS mapping spectra of the coatings A and B after 25 days of immersion in a 3 wt. % NaCl solution. Figure 3a shows that the zinc particles were completely isolated by the epoxy resin. The diameters of the zinc particles were in the range of 10 μm to 20 μm. Many intact spherical zinc particles were observed in the coating even after 25 days of immersion, as clearly shown in the zinc element EDS mapping (see Figure 3b). The presence of a large number of isolated zinc particles could be attributed to the inactive cathodic protection process. However, as shown in the Figure 3d,e, in the presence of 0.6 wt. % of single-layer Gr, almost all of the zinc particles were more uniformly corroded. The zinc particles were in direct contact with each other and gradually joined instead of isolated. 

The results showed clearly that the single-layer Gr changed the distribution of the zinc in coating. In the absence of the single-layer Gr, zinc oxides/hydroxides distributed in an isolated manner in the coating (Figure 2a and Figure 3b), resulting to little effective cathodic protection for the steel substrate. In the presence of Gr, however, zinc elements were detected at the coating-steel interface instead of assembling on the surface (Figure 2b and Figure 3e). Compared with freshly prepared coating B (Figure S3), Zn-containing species jointed together with increasing of particle size. This behavior can be associated with the “bridge” effect of the single-layer Gr, which facilitated charge transfer process between zinc particles and steels. Thus, the zinc oxide particles moved towards the steel under galvanic action and gravity, in order to provide sacrificial action to the steel substrate.

The XPS high-resolution spectra for Zn 2p3/2 are shown in Figure 3c,f, which characterized the elemental and chemical composition on the coating surface after immersion in the 3 wt. % NaCl solution for 25 days. The peaks at 1021.7 eV, 1022.0 eV, and 1022.4 eV can be attributed to the binding energies of Zn_5_(CO_3_)_2_(OH)_6_, metallic zinc and ZnO, respectively [22]. The mechanism of the zinc dissolution in a neutral solution can be described as follows:

O_2_ + 2H_2_O +4e^−^ → 4OH^−^, (1)

Zn − 2e^−^ → Zn^2+^, (2)

Zn + 2OH^−^ → Zn(OH)_2_ + 2e^−^. (3)

Then, carbon dioxide was adsorbed from the environment into the sample along with Zn(OH)_2_, resulting in the formation of Zn_5_(CO_3_)_2_(OH)_6_ and ZnO, as shown in the following reaction [23]:

6Zn(OH)_2_ + 2CO_2_ → Zn_5_(CO_3_)_2_(OH)_6_ + ZnO + 3H_2_O, (4)

In the presence of Gr, the relative content of zinc oxide increased obviously, indicating that accelerated corrosion of zinc occurred with the addition of the Gr, due to the extended cathodic protection.

**Figure 4:**
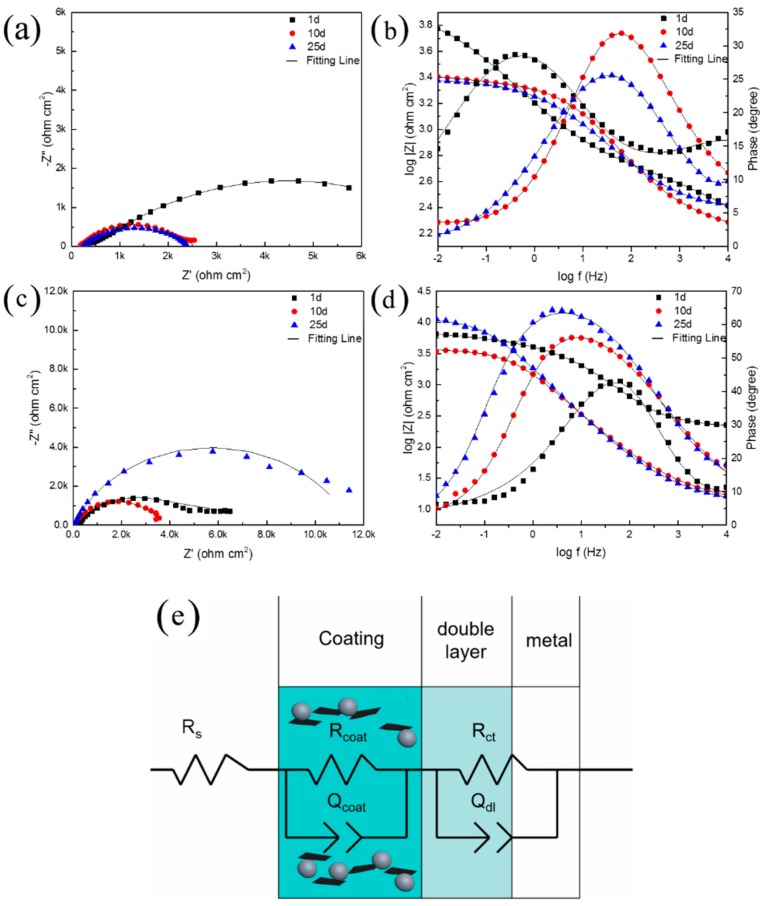
Nyquist and Bode diagrams measured on the coated sample (**a**,**b**) ZRPs (**c**,**d**) Gr0.6-ZRPs of immersion in a 3 wt. % NaCl solution with (**e**) equivalent circuit and model.

Figure 4 shows the EIS Nyquist and Bode plots for the coated samples A and B at different immersion times (1 day, 10 days, 25 days) in the 3 wt. % NaCl solution. Figure 4 shows that the electrochemical impedance curves for the two samples both consist of two incomplete semicircles, indicating two time constants for the coated samples. The two time constants (or loops in the Nyquist plot), which were distinguished at low frequency and high frequency, were attributed to the charge transfer processes at the electrolyte/steel substrate and electrolyte/coating interfaces, respectively. An electrochemical equivalent circuit composed of the solution resistance (*R*_s_), charge transfer resistances of the steel specimens (*R*_ct_), resistance of the coating or film (*R*_coat_), and constant phase elements (CPE, *Q*), i.e., *R*_s_(*R*_ct_*Q*_dl_)(*R*_coat_*Q*_f_) was utilized to simulate the experimental data. *Q* was used to replace the double-layer capacitance in order to justify the actual surface conditions of the electrode. The impedance of *Q* is defined by:

*Z*(*j*ω) = (*Y*_0_)^−1^(*j*ω)^−n^, (5)

where *Y*_0_ is the *Q* constant, *j* is the imaginary unit, *n* is the *Q* power (0 ≤ *n* ≤1), and ω is the angular frequency (ω = 2π*f*, *f* is the frequency). For *n* = 1, *Q* is the pure capacitance. For a capacitance element, the deviation of *n* from the unit is due to the heterogeneity effect.

The fitted impedance parameters are listed in Table 2. For Coating A, the film resistance (*R*_coat_) decreased from 6630 Ω·cm^2^ to 3345 Ω·cm^2^ after 10 days, and then remained at this level. This behavior can be attributed to the permeation of the electrolyte with a relatively high porosity in the presence of zinc particles, which was consistent with the surface morphology shown in Figure 1a. However, with the addition of the 0.6 wt. % Gr, the coating resistance (*R*_coat_) decreased slightly after 10 days of immersion and then increased considerably. For example, the value of *R*_coat_ decreased from 6630 Ω·cm^2^ to 6100 Ω·cm^2^ during the first 10 days of immersion. After that, the *R*_coat_ increased again, and finally reached 11,200 Ω·cm^2^ on the 20th day of immersion.

We identified two different stages in the immersion test based on the trend in the impedance data for Coating B. During the initial stage (before the tenth day), electrolyte penetration took place inside the pores of the coating, resulting in a decrease in *R*_coat_. In the second stage, after 10 days of immersion, there was a significant increase in the coating resistance. The cathodic protection was especially active in this stage, resulting in the formation of Zn_5_(CO_3_)_2_(OH)_6_ and ZnO, which took place in the coating, as shown in XPS analysis (Figure 3f). These products fill in pores and reduce the amount of electrolyte that reaches the steel surface, increasing impedance semicircle over time.

**Figure 5:**
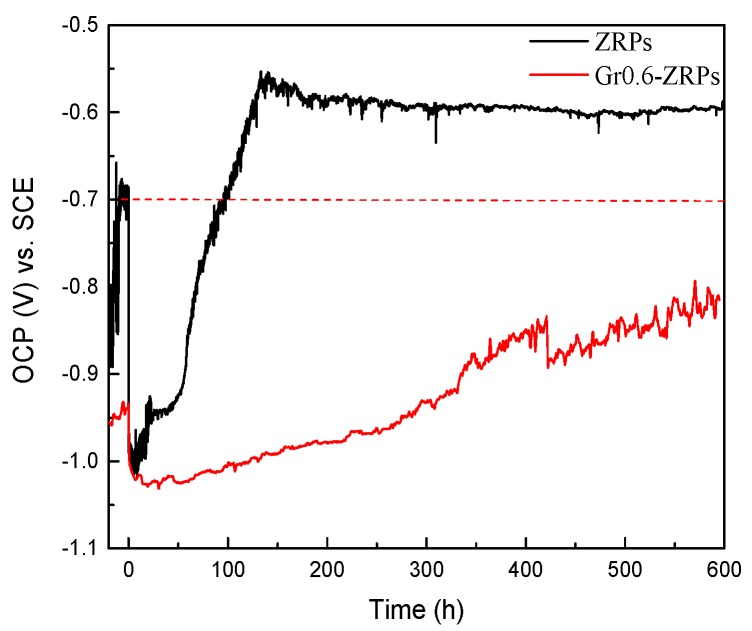
OCP-time measurement of ZRPs and Gr0.6-ZRPs in a 3.5% NaCl solution during 600 h of immersion.

The OCP-time measurements were conducted to confirm the effect of single-layer Gr on the cathodic protection of coatings. The corrosion potential of zinc and carbon steel in a 3.5 wt. % NaCl solution is approximately −1.05 and −0.55 V (vs. SCE), respectively. The mixed interface potential was dependent on the active surface area between zinc particles and steel. More zinc particles in that electrical contact with steel cause a negative shift of mixed interface potential. As shown in Figure 5, the more negative interface potential of Coating B can be attributed to the “bridge” effect of single-layer Gr. 

**Figure 6:**
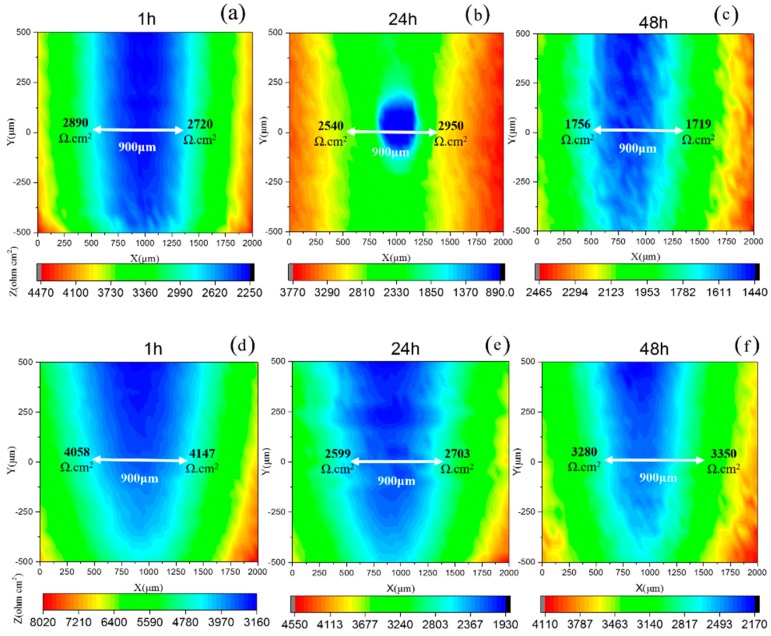
LEIS results for (**a**–**c**) ZRPs and (**d**–**f**) Gr0.6-ZRPs with an artificial scratch immersed in 0.005 M NaCl during 48 h.

Since the cathodic protection was enhanced by the single-layer Gr, LEIS measurements were performed on the scratch-coated steel specimens. Artificial damage (0.2 mm × 1 mm) was induced in order to investigate the cathodic protection performance of the coating. Then samples with the artificial defect were exposed to a 5 mM NaCl solution for 48 h. The LEIS spectra for Coating A are given in Figure 6a–c; here, the local impedance in the scratched area decreased continuously during the 48 h of exposure, from 2890 Ω·cm^2^ to 1756 Ω·cm^2^. The dissolution process of the steel was related to this behavior, since a pitting spot was identified after 24 h of immersion. However, with addition of 0.6 wt. % Gr, the local impedance for Coating B in the scratched area decreased from 4358 Ω·cm^2^ to 2599 Ω·cm^2^ in the initial 24 h of immersion and increased considerably to 3353 Ω·cm^2^ after 48 h, as shown in Figure 6d–f. It is important to note that the damage area shrink obviously, which is attributed to the fact that the zinc oxide formed on the damage coating. It indicates that the sacrificial protection is a predominant mechanism with addition of the 0.6 wt. % single-layer Gr, which could be due to the increase in the electrical connection between the zinc particles and steel substrate. 

**Figure 7:**
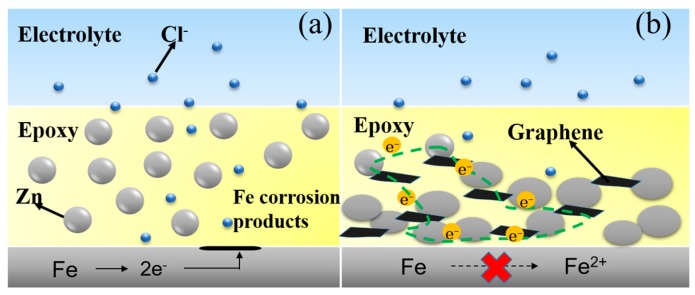
The schematic representation of (**a**) ZRPs and (**b**) Gr0.6-ZRPs during immersion in the electrolyte.

Figure 7 shows a diagram of the effect of single-layer Gr on the corrosion protection of the zinc-rich epoxy primer on steel. In the absence of the Gr, the zinc particles (with spherical geometry) are distributed in the resin, and there is almost no contact among the particles or with the carbon steel surface (Figure 5a). Chloride ions and oxygen molecules will permeate into the coating and lead to the rapid corrosion of the steel under these conditions. In the presence of the 0.6 wt. % modified Gr, sacrificial protection is the predominant mechanism, which could be due to the increase in the electrical connection between the zinc particles and steel substrate (Figure 5b). During the immersion time, a layer of zinc corrosion products, rather than iron corrosion products, formed on the steel surface. Additionally, a moderate amount of the single-layer Gr increased the electrical conductivity of the coating, which facilitated the charge transfer processes between the zinc particles and steel substrate.

Effects of addition of graphene on corrosion mechanism of ZRP have been reported in several papers [17,18], and it is general accepted that a certain content of Gr would facilitate improving the cathodic protection performance of the ZRP coating. Herein, we innovatively focused on the effect of single-layer Gr on the mechanism of corrosion protection of the defect area. Compared to the ZRPs coating, OCP of the Gr0.6-ZRPs coating in Figure 5, maintained in the cathodic protection region for the whole immersion testing time. The strong and stable cathodic protection performance of the Gr0.6-ZRPs coating could be attributed to the more effective activation of zinc particles around the artificial defects. For the ZRPs coating, the zinc particles with a spherical geometry were distributed in the resin where there was no connection tunnel between the particles and the carbon steel surface, as illustrated in Figure 7a. In this condition, the presence of Cl^–^ and oxygen permeate into the coating and lead to rapid corrosion of the steel at the defect area. The presence of 0.6 wt. % Gr, working as an electric connection bridge, facilitated the transfer of the electron from zinc to the substrate, which resulted in the enhancement of the sacrificial efficiency. The results of LEIS of the selected area in Figure 6 and those of EIS in Figure 4 indicated that the impedance increased with the immersion time for the Gr0.6-ZRPs and that the values of impedance were larger than that of the ZRPs coating. The sacrificial dissolution of zinc particles took place, resulting in the formation of ZnO and/or Zn_5_(CO_3_)_2_(OH)_6_ particles. Formation of zinc corrosion products would be beneficial for improving physical barrier properties [5,15]. Additionally, it was reported that the presence of 2D nanosheets in coatings, e.g., graphene [14], typically extends the diffusion path of the electrolyte solution, resulting in the improvement of coating physical barrier.

## 4. Conclusions

Corrosion protection mechanisms were examined with zinc-rich epoxy primers with and without single-layer Gr in a 3 wt. % NaCl solution. The SEM and XPS results indicated that the addition of Gr accelerated the electrochemical reaction of zinc particles and steel substrate. The zinc corrosion products mainly included ZnO and Zn_5_(CO_3_)_2_(OH)_6_ that formed on the steel, resulting in increasing the impedance semicircle over time, which could be attributed to the barrier protection effect. LEIS and OCP-time measurements indicated that Gr promoted cathodic protection of the ZRPs, in the presence of artificial scratch damage on the surface of coatings. Therefore, the 0.6 wt. % single-layer Gr can provide both barrier and cathodic protection for ZRPs.

## Figures and Tables

**Table 1:** Specification of different coatings.

Parameter	Zinc (wt. %)	Gr (wt. %)	Dry Film Thickness (μm)
ZRPs	80	0	120 ± 9.6
Gr0.6-ZRPs	80	0.6	120 ± 8.5

**Table 2:** Electrochemical impedance parameters fitted from the measured impedance plots in Figure 4.

Samples/Day	*R*_s_ (Ω·cm^2^)	*C*_coat_ (Ω^−1^·cm^−2^·s^n^)	*R*_coat_ (Ω·cm^2^)	*C*_dl_ (Ω^−1^·cm^−2^·s^n^)	*R*_ct_ (Ω·cm^2^)
ZRPs-1 d	0.405	1.36 × 10^−4^	6630	3.12 × 10^−4^	770
ZRPs-10 d	0.842	4.16 × 10^−5^	3350	2.23 × 10^−4^	739
ZRPs-25 d	0.997	5.43 × 10^−5^	3030	8.99 × 10^−5^	220
Gr0.6-ZRPs-1 d	0.702	3.47 × 10^−4^	6630	2.45 × 10^−5^	349
Gr0.6-ZRPs-10 d	1.08	2.50 × 10^−3^	6100	1.46 × 10^−4^	320
Gr0.6-ZRPs-25 d	0.734	2.61 × 10^−3^	11200	8.02 × 10^−3^	983

## Supplementary Materials

The following are available online at http://www.mdpi.com/2073-4360/10/6/591/s1, Figure S1: Images of modified graphene dispersed in three solutions: water, ethanol and dimethylbenzene. Figure S2: TEM image of a single-layer graphene. Figure S3: The cross sectional (a) SEM image and (b) elemental map for the freshly coated samples of Gr0.6-ZRPs before immersion in a 3 wt. % NaCl solution.
