# Supplementary material for: Promoting Barrier Performance and Cathodic Protection of Zinc-Rich Epoxy Primer via Single-Layer Graphene
Source: Polymers (Basel). 2018 May 28;10(6):591. doi: 10.3390/polym10060591 (PMC6404031; doi:10.3390/polym10060591)

SUPPLEMENTAL FIGURES AND FIGURE LEGENDS

Supplementary Figure S1: Images of modified graphene dispersed in three solutions: water, ethanol and dimethylbenzene.

Supplementary Figure S2: TEM image of a single-layer graphene.

Supplementary Figure S3: The cross sectional (a) SEM image and (b) elemental map for the freshly coated samples of Gr0.6-ZRPs before immersion in a 3 wt. % NaCl solution.

Supplementary Figure S1

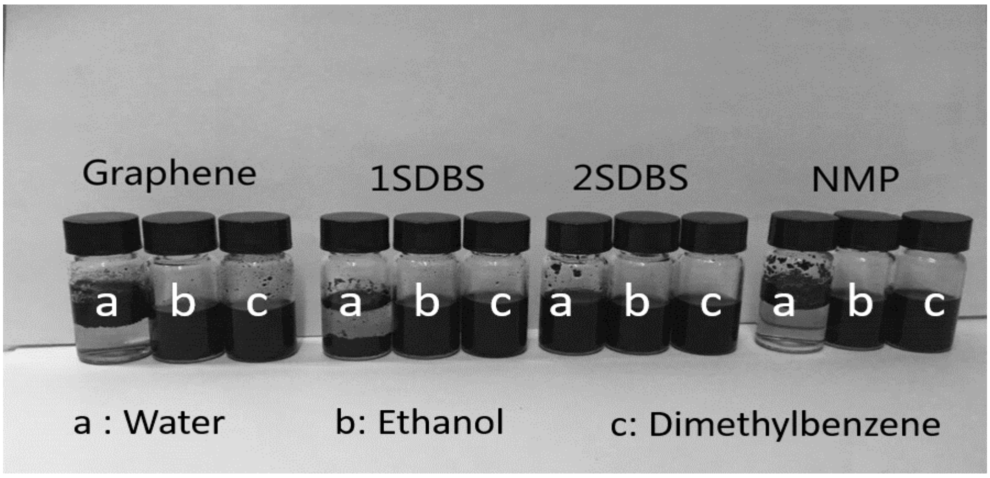

Supplementary Figure S2

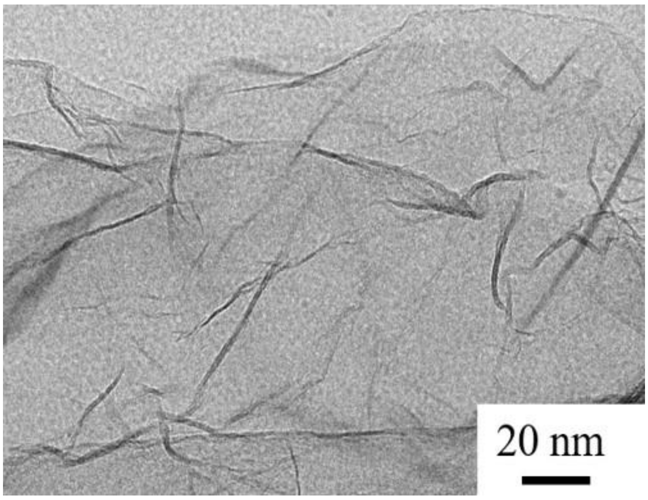

Supplementary Figure S3

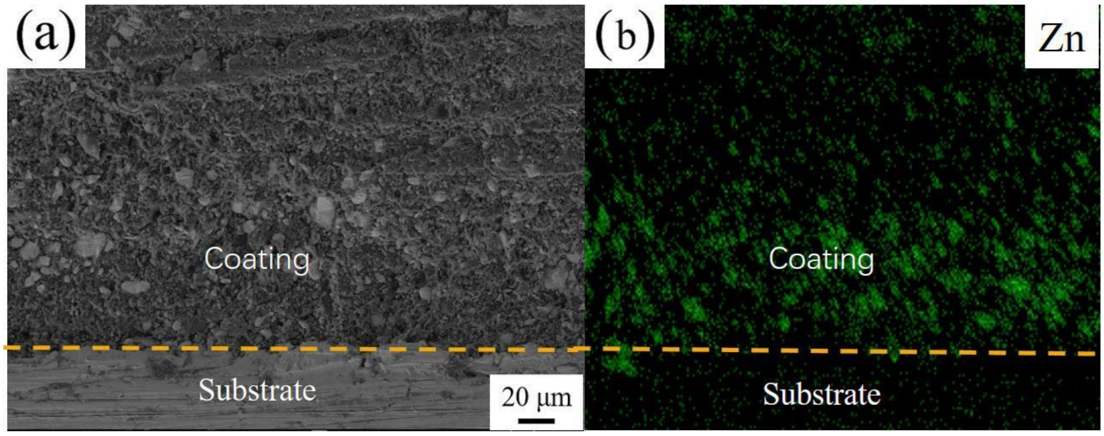

Supplement: Supplementary file 1 [file polymers-10-00591-s001.pdf]
